# Characterization analysis of Rongchang pig population based on the Zhongxin-1 Porcine Breeding Array PLUS

**DOI:** 10.5713/ab.23.0049

**Published:** 2023-06-26

**Authors:** Dong Leng, Liangpeng Ge, Jing Sun

**Affiliations:** 1 Chongqing Academy of Animal Science, Chongqing 404100, China; 2 Farm Animal Genetic Resources Exploration and Innovation Key Laboratory of Sichuan Province, Sichuan Agricultural University, Chengdu, 611130, China; 3 Key Laboratory of Pig Industry Sciences, Ministry of Agriculture, Chongqing 404100, China; 4 National Center of Technology Innovation for Swine, Chongqing 404100, China

**Keywords:** Genetic Distance, Genetic Diversity, Inbreeding Coefficient, Single Nucleotide Polymorphism (SNP) Chip, SPF Rongchang Pigs

## Abstract

**Objective:**

To carry out a comprehensive production planning of the existing Rongchang pig population from both environmental and genetic aspects, and to establish a closed population with stable genetic diversity and strict pathogen control, it is necessary to fully understand the genetic background of the population.

**Methods:**

We genotyped 54 specific pathogen free (SPF) Rongchang pigs using the Zhongxin-1 Porcine Breeding Array PLUS, calculated their genetic diversity parameters and constructed their families. In addition, we also counted the runs of homozygosity (ROH) of each individual and calculated the value of inbreeding coefficient based on ROH for each individual.

**Results:**

Firstly, the results of genetic diversity analysis showed that the effective population size (N_e_) of this population was 3.2, proportion of polymorphic markers (P_N_) was 0.515, desired heterozygosity (H_e_) and observed heterozygosity (H_o_) were 0.315 and 0.335. H_o_ was higher than H_e_, indicating that the heterozygosity of all the selected loci was high. Secondly, combining the results of genomic relatedness analysis and cluster analysis, it was found that the existing Rongchang pig population could be divided into four families. Finally, we also counted the ROH of each individual and calculated the inbreeding coefficient value accordingly, whose mean value was 0.09.

**Conclusion:**

Due to the limitation of population size and other factors, the genetic diversity of this Rongchang pig population is low. The results of this study can provide basic data to support the development of Rongchang pig breeding program, the establishment of SPF Rongchang pig closed herd and its experimental utilization.

## INTRODUCTION

Rongchang pig is not only one of the eight excellent pig breeds in the world, but also one of the three excellent local pig breeds, which has been listed as a national class I protected breed resource in China [1]. As a typical fatty pig breed in southwest China, it has been reared for more than four decades and is mainly produced in Rongchang District of Chongqing. Rongchang pigs are an ideal experimental animal model with excellent characteristics such as high adaptability, good disease resistance, and stable genetic performance [2–4]. Compared to large pigs, miniature pigs can reach sexual maturity earlier, while Rongchang pigs are smaller than three-breed crossbred sows, but larger than experimental miniature pigs. They have an earlier puberty with an average age of 106.1±18.7 days and an average weight of 26±10.2 kg, making them an ideal pig breed for pure line breeding and establishing specific pathogen free (SPF) populations [1].

The SPF animals are more suitable than conventional animals for establishing animal models because they do not carry major potential infections and pathogens that interfere with scientific research. SPF animals can be used for a variety of scientific experiments, including vaccine manufacturing, bioassays, human tumor xenografts, preclinical pharmacology studies, and carcinogenicity testing [5]. The 54 SPF Rongchang pig population used in this study is free of bacterial enteric pathogens, swine viruses, ova and parasites, and mycoplasma hyopneumoniae. However, the genetic background of this population remains unknown. In recent years, our team has also carried out some important work in high microbial grade (germ-free and SPF) laboratory-housed pig resources [6–8]. But these preliminary studies focused more on the control of swine pathogens and biosafety, and the study on population genetic control is just starting. Genetic diversity within a breed is influenced by genetic drift, selection, migration, and mutation. Genetic diversity is not static. Within breeds, it is threatened by intensive selection for a few traits and an increase in the genetic relationship between animals, which leads to inbreeding and its negative effects [9]. Therefore, maintaining genetic diversity is important because it largely determines the likelihood of selection, and inbreeding is avoided [10].

To develop and utilize Rongchang pig resources in swine diseases prevention and control, identification or screening experiments of biological products and chemicals, it is necessary to make a comprehensive production plan for laboratory pigs from both environmental and genetic aspects, to establish a closed population with stable genetic diversity and strict pathogen control. This study intends to carry out the genetic analysis by single nucleotide polymorphism (SNP) chips based on the Zhongxin-1 Porcine Breeding Array PLUS, to reveal the important genetic parameters of this population, and to lay the foundation for the development and implementation of future breeding programs.

## MATERIALS AND METHODS

### Experimental animals and its care

The Experimental Pig Engineering Center of Chongqing Institute of Animal Science (Rongchang, Chongqing, China) provided the experimental pigs for this study. All animal experiments were performed in accordance with the Regulations on the Administration of Laboratory Animals (Ministry of Science and Technology, Beijing, China; revised June 2004). The Ethics Committee of Chongqing Institute of Animal Science approved this study under Permit No. 2020012B.

### Sample collection and SNP genotyping

In this study, we selected 54 SPF Rongchang pigs, including 6 males and 48 females. Genomic DNA was extracted from ear tissues using a routine phenol/chloroform method and was diluted to a final concentration of 50 ng/μL [11]. The total amount of DNA after mass inspection was greater than 1 μg, and the absorbance 260/280 ratio of DNA was between 1.7 and 2.1.

We genotyped all pigs following the manufacturer’s protocol using the SMIC One chip [12], an Illumina genome-wide SNP chip, and detected 57,466 SNP loci in total. After that, we used PLINK (V1.90, Shaun Purcell) [13] for quality control of the genotype data, and only the SNP loci meeting the quality control conditions were retained for subsequent analysis. In this study, we only considered SNPs on autosomes and required that both SNP detection rates (call rates) and individual detection rates be greater than or equal to 90%. Quality control conditions for polymorphic markers (P_N_) analysis also included a significance threshold of 0.000001 was used for Hardy Weinberg equilibrium test. Quality control conditions for effective population size (N_e_), desired heterozygosity (H_e_), observed heterozygosity (H_o_), ***G*** matrix, identity by state (IBS) genetic distance, and cluster analysis also included a significance threshold of 0.000001 was used for Hardy Weinberg equilibrium test, and minor allele frequency (MAF) greater than or equal to 0.01. Finally, a total of 50,196 SNP loci were screened for P_N_ analysis, 29,688 SNP loci for N_e_, H_e_, H_o_, ***G*** matrix, IBS genetic distance, and cluster analysis, and 50,359 SNP loci for runs of homozygosity (ROH)-based inbreeding coefficient (*F_ROH_*) analysis.

### Genetic diversity

In this study, we used SNeP (V1.1, Mario Barbato) [14], PLINK and self-programmed R script to estimate several genetic diversity parameters in Rongchang pigs, such as N_e_, P_N_, H_e_, and H_o_.

N_e_ refers to the ideal population content with the same gene frequency variance or the same inbreeding coefficient increment (heterozygosity decay rate) as the actual population [15], which is usually estimated based on the level of linkage disequilibrium (LD) of the population [14]. We used the calculation method proposed by Herrero-Medrano et al [16] and Sved for the analysis [17], which is as follows.


$$N_{\text{e}}=\frac{1}{4c}\times \left(\frac{1}{{\text{r}}^{2}}-1\right)$$

Where r2 is the degree of linkage between SNP loci and c is the molar root distance between SNP loci in centimorgan.

PN refers to the proportion of loci that exhibit polymorphism in the target population to the total loci. A larger PN shows more polymorphic markers in the population, and conversely, indicates a greater likelihood of extinction of certain favorable alleles. We first calculated the MAF for each locus using PLINK and then calculated the PN using a self-programmed R script. We calculated the PN according to the following equation.


$$P_{N}=\frac{M}{N}$$

Where M is the number of bits that exhibit polymorphism and N is the total number of all nucleotide loci detected bits.

H_e_ refers to the probability of heterozygosity at any locus in the population; H_o_ refers to the number of individuals in the population that are heterozygous at a locus as a proportion of the total number of individuals [15]. When the observed heterozygosity is lower than the expected heterozygosity, it is assumed that the population might have experienced selection or increased inbreeding events. Similarly, a greater observed heterozygosity than expected might indicate a possible exposure of that population to some new exotic genetic resources. We analyzed the observed heterozygosity and expected heterozygosity using the method proposed by Sun et al [15] and Nei, which is as follows.


$$\begin{array}{l} H_{o}=\frac{1}{N}\sum_{k=1}^{N}\frac{H_{k}}{n}\\ E(H)=\frac{2n}{2n-1N}\frac{1}{N}\sum_{k=1}^{N}\left(1-\sum {P^{2}}_{ki}\right) \end{array}$$

Where n is the total number of individuals in the population, N is the total number of loci, H_k_ is the number of heterozygous individuals at locus k, and P_ki_ is the frequency of allele i at locus k.

### Phylogenetic analysis and cluster analysis

First, we used PLINK to calculate IBS genetic distances, and the results were used for subsequent phylogenetic tree construction. IBS refers to the DNA fragment identical by descent in two or more individuals, and these DNA fragments have the same base sequence. Subsequently, we used the ***G*** matrix (V2, VanRaden) [18] to calculate the kinship values and the ComplexHeatmap (V2.12.1, Zuguang Gu) [19] to plot the heatmap. Finally, cluster analysis was performed by Mega X (V10.0, Sudhir Kumar) [20] to classify 54 Rongchang pigs into different family groups. The ***G*** matrix was a genomic relationship matrix constructed using genome-wide markers based on the genomic relationship ***G*** matrix construction method proposed by VanRaden [18]. Since the pedigree information of a conserved population is usually not recorded, ***G*** matrix is suitable for calculating the genetic relationship, which is as follows.


$$G=\frac{ZZ^{\prime }}{2\sum p_{i}(1-p_{i})}$$

Where Z and Z′ are matrices containing the content of sub-alleles per sample, per locus (after centralization), and P_i_ is the frequency of the i-th allele.

Genomic kinships between two individuals were calculated using the method proposed by VanRaden. The algorithm states that the genomic kinships of individual j and individual k are obtained by dividing G_jk_ by the square root of the product of G_jj_ and G_kk_, which is as follows.


$${KS}_{\text{jk}}=\frac{G_{jk}}{\sqrt{G_{jj}G_{kk}}}$$

Where KS_jk_ is the genomic affinity of individual j and individual k, G_jk_ is the element value of individual j and individual k in the ***G*** matrix, G_jj_ is the diagonal element value of the ***G*** matrix of individual j, and G_kk_ is the diagonal element value of the ***G*** matrix of individual k.

We constructed phylogenetic trees of all samples based on the IBS genetic distance matrix using the neighbor-joining method. Based on the analysis, it can be roughly inferred which Rongchang pig individuals are closer in blood relationship and the samples originate from the same family group.

### Inbreeding coefficient analysis

The ROH was first detected using the sliding window method in PLINK, and the conditions defined as ROH included: 1 SNP per 1,000 kb, two consecutive SNPs with an interval of no more than 1,000 kb, length greater than 1,000 kb, and containing more than 30 SNPs. The maximum number of heterozygous and missing SNP sites in the slider window was one, the size of the slider window was 50 SNPs, and the proportion of completely pure sliders containing a particular SNP was at least 5% [13]. The ROH length was then calculated for each sample. Finally, the ROH-based inbreeding coefficient (*F_ROH_*) was obtained by calculating the total length of ROH segments in individuals as a proportion of the total autosomal genome length [21]. *F_ROH_* was proposed by McQuillan et al [22] and is defined as the length of autosomes covered by ROHs divided by the total autosomal total length. The longer the total length of ROHs in an individual, the higher the inbreeding coefficient of that individual, which is as follows.


$$F_{ROH}=\frac{\sum_{k}Length({ROH}_{k})}{L}$$

Where k is the number of ROH in the individual and L is the length of the autosomal genome covered by the analyzed data chip (2,450,462.292 kb).

The average inbreeding coefficient value of the population was obtained by summing the inbreeding coefficient values of all individuals and dividing by the total number of individuals, which is as follows.


$$F_{ROH}=\sum_{i=1}^{N}\frac{F_{ROHi}}{N}$$

*F_ROHi_* is the inbreeding coefficient value of the i-th individual, and N is the total number of individuals.

## RESULTS AND DISCUSSION

### Genetic diversity analysis

Genetic diversity mainly refers to the degree of genetic variation between different varieties and within the same variety, which is an important reference for the protection of genetic resources. Assessment of the genetic diversity of the current population based on genome-wide data provides a better understanding of the current conservation status of the population. In this study, we analyzed the genetic diversity of 54 Rongchang pigs, and the N_e_ of this population was 3.2. N_e_ is the fundamental factor that affects the rate of inbreeding (ΔF), and the larger Ne, the smaller ΔF. Numerous investigators reported N_e_ of 50 as a threshold necessary to avoid the negative impact of inbreeding on pig populations, while a population size of 500 pigs is necessary to maintain the genetic diversity and evolutionary potential of the population for several generations [23–25]. Meuwissen and Woolliams recommended that the N_e_ value be maintained between 31 and 250 animals for good population fitness [26]. A recent study, which analyzed the genetic diversity and population structure of 46 Xinjiang Altay white-headed cattle, found that the population had a N_e_ of only 2.4 [27]. Therefore, the authors made a detailed plan to ensure the long-term protection of Altay white-headed cattle genetic resources. This suggests that a rational selection and mating scheme should be developed to prevent the loss of independent consanguinity relations in the alternation of generations, thus maintaining the reproduction and expansion of the Rongchang population and further improving the N_e_ value. It is also possible to increase the germplasm exchange between different Rongchang breeding farms and actively introduce new germplasms, especially new boar germplasms.

P_N_ represents the probability of presenting polymorphic markers loci in the population, the population P_N_ is 0.515. This indicates that the population is relatively rich in the number of polymorphic marker loci and that the risk of extinction of favorable alleles is low. H_e_ and H_o_ are 0.315 and 0.335, respectively, and H_o_ is higher than H_e_, indicating that the selected loci were all well heterozygous. The low percentage of homozygous individuals in this population indicates that the population may be mixed with germplasms of other breeds and needs to continue purification. This may also be strongly related to the short breeding history of this population. In addition, it may be difficult to effectively increase population size and expand genetic diversity with traditional breeding conservation methods. Further research of the genetic structure of populations at the molecular level will facilitate population conservation efforts [28]. For example, whole genome re-sequencing techniques are used to discover variant loci and candidate genes related to influencing important economic traits, which can then be verified in the next step, thus accelerating the pace of molecular breeding in animals [29].

### Molecular kinship analysis

**Figure 1:**
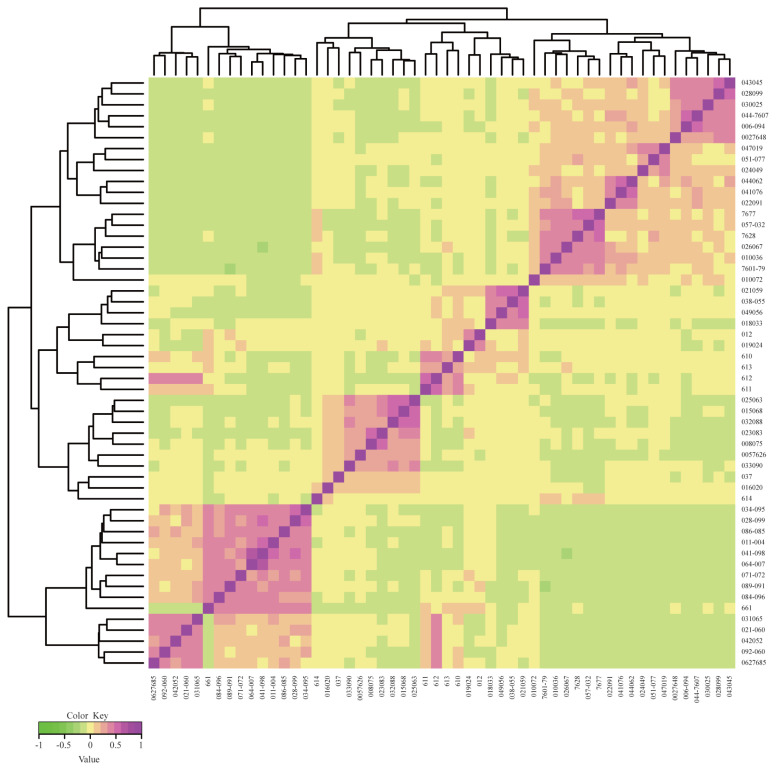
***G*** matrix heatmap of a conserved population of Rongchang pigs using ***G*** matrix and ComplexHeatmap. Each small square represents the value of the relationship between any two individuals. The larger the value, the closer the color of the square is to red, and the closer the relationship between two individuals.

Based on the kinship analysis (Figure 1), we identified approximately four genetic clusters among 54 pigs. Relationship coefficient is an important indicator for evaluating the relationship between individuals within a population. With a non-inbred common ancestor, relationship coefficient of half-sib correlation is 0.25 and that of full-sib correlation is 0.5. Our results showed that the average relationship coefficient of Rongchang pigs was 0.106, with about 7.99% of individuals having a relationship coefficient between 0.25 and 0.5, about 21.53% of individuals having a relationship coefficient between 0.125 and 0.25, and about 70.49% of individuals having a relationship coefficient less than or equal to 0.125. This indicates that the number of half-sibs and full-sibs in this population is small and that most individuals are genetically distant from each other. At present, the pedigree data still represent the base for analyses of genetic diversity [30]. Complete records of pedigree information are advantageous for the analysis of genetic parameters such as population structure and inbreeding levels [31]. A recent study estimated parameters related to the population structure and genetic diversity of the Simmental breed based on pedigree information from 77,553 Simmental beef cattle [32]. In addition, there are studies that analyzed population structure and inbreeding levels based on pedigree information of 9,170 Polish red bulls [33]. Compared to our study, they have more abundant production data for calculating metrics such as Ne and inbreeding levels. Due to the lack of pedigree data, we could not determine the exact kinship of these 54 Rongchang pigs [34]. Therefore, in the follow-up work, we need to record the pedigree information of the conservation population in detail to provide favorable information for the subsequent population expansion and the establishment of a closed SPF pig colony.

### Group family construction

**Figure 2:**
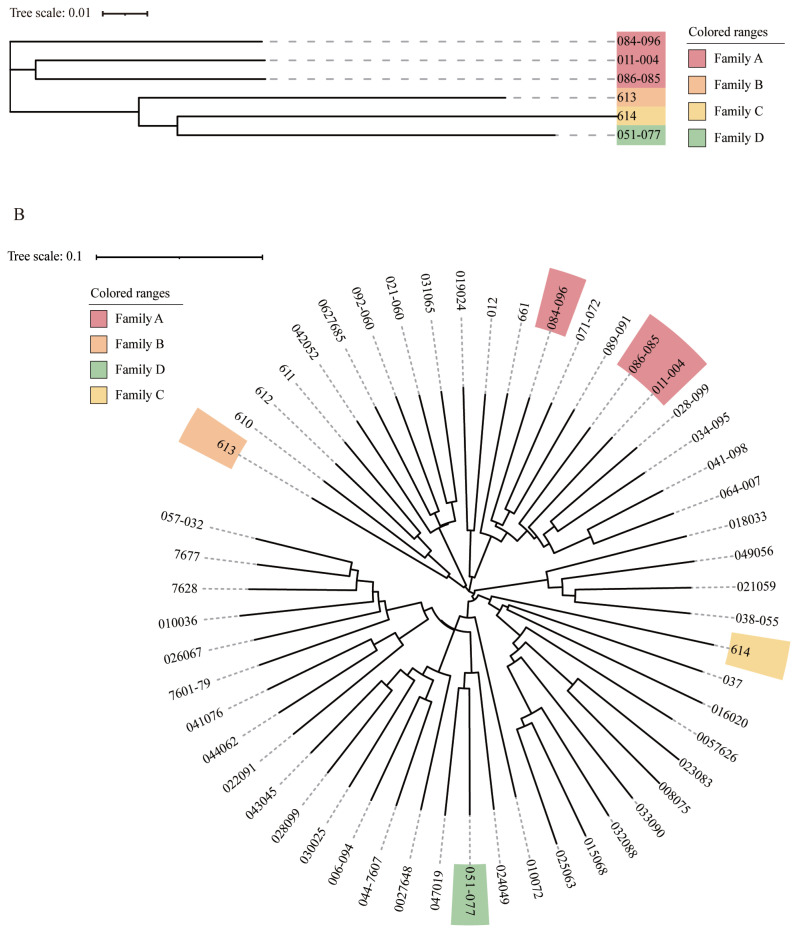
Family construction of the Rongchang pig breeding conservancy population. (A) Evolutionary tree of boar samples in the conserved population. Samples labeled with the same color are evaluated as the same family. All boars grouped into 4 families. (B) Phylogenetic tree of 54 Rongchang pigs, in which all boar samples are marked by color, with different colors representing different families.

In view of the importance of boars to the entire conservation population, cluster analysis was performed using six boars to judge the kinship between them. These boars were divided into four families according to the classification criteria that the molecular kinship between boar is greater than or equal to 0.1. Combined with the results of the kinship analysis, it was found that the existing boars can be roughly divided into four families A, B, C, and D (Figure 2A). Family A includes three boars; B, C, and D each include one boar. Currently, the number of boars in the entire herd is small, with family A including three boars and each of the three families B, C, and D including only one boar. Therefore, in the subsequent population conservation process, attention should be paid to the selection and breeding of offspring. Sperm freezing technology can be used to construct a breeding pig gene bank to reduce the cost of conservation, improve the protection efficiency, and avoid causing loss of consanguinity.

Twenty-three sows were classified into four constructed boar families according to the distance of kinship to the boars of the different families. The other 25 sows had kinship coefficients less than 0.1 with all boars and were therefore divided into a separate family (Figure 2B). Boars and sows classified into the same family had closer kinship and they had a higher probability of being from the same ancestor. In addition, the number of boars and sows as well as the degree of inbreeding were unbalanced among the different families. Previous studies have shown that the population generated by retaining each family line in the same proportion, the upper population provided the same proportion of the number of retained seeds to the lower population with zero variance [35]. This helps to improve the N_e_ of the population and is effective in slowing down the generational growth of the inbreeding coefficient. Therefore, we should pay attention to scientific selection and optimize population structure in the future breeding conservation process.

### ROH based inbreeding coefficient analysis

**Figure 3:**
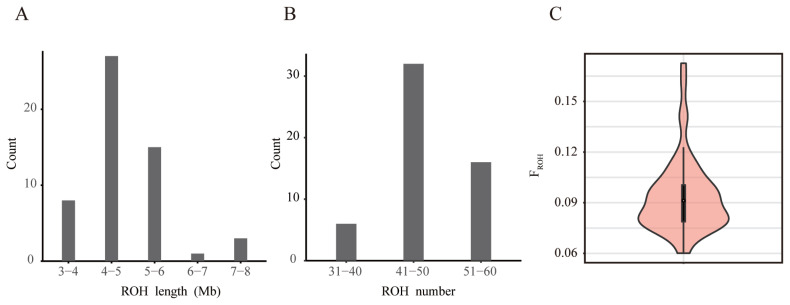
Estimation of inbreeding degree of Rongchang pig breeding population using ROH (A) Distribution of ROH lengths in the population of conservation. (B) Distribution of ROH numbers in the population of conservation. (C) The distribution of FROH is shown using the violin plot. The white point in the center represents the median of the population *F_ROH_*, and the upper edge of the black box is the upper quartile and the lower quartile of the group *F_ROH_*. The width of the violin diagram represents the probability density distribution of the population *F_ROH_*. ROH, runs of homozygosity.

The evaluation of inbreeding coefficients can provide a basis for the selection, protection, and utilization of population. The length and frequency of ROH can reflect population history. A long ROH can indicate kinship that occurred in the more recent past; the more such segments, the higher the likelihood of inbreeding within the lineage. Short ROHs, on the other hand, indicate kinship that occurred at a more distant time, and existing genealogies usually no longer explain these kinships [9]. Currently, the number of individual ROHs in the Rongchang pig population ranges from 35 to 60, and the length ranges from 3 to 8 Mb. Most individuals have ROH lengths of 6 Mb or less, indicating a long history of inbreeding (Figure 3A–B; Supplementary Table S1). This may be due to the different generations and selection strategies used by the conserved farm, as well as the different degrees of selection pressure on the traits of these lines [36].

*F_ROH_*, defined as the percentage of the genome covered by ROH, is considered to be an indicator of the recent inbreeding history of a population and has also shown good correlation with the pedigree inbreeding coefficient [37]. A recent study showed that breeds with high genetic diversity exhibit low *F_ROH_*, demonstrating the reliability of using *F_ROH_* to indicate inbreeding [38]. Calculating the *F_ROH_* was more accurate than inbreeding coefficients estimated from lineage data (*F_PED_*) for estimating autozygosity and detecting past and recent inbreeding effects [39]. The better results of *F_ROH_* suggest that in the absence of pedigree information, *F_ROH_* can be used to infer information about the history and inbreeding levels of a population. The N_e_ of the Rongchang pig population was only 3.2, which implies that there may be a more serious inbreeding situation. However, according to our calculations, the average *F_ROH_* of this population was 0.09, which is not very high (Figure 3C; Supplementary Table S1). This could be because the population is not a closed population with a long breeding history. It is also possible that this is because germplasm resources from other Rongchang pig populations were introduced during the previous mating work. In order to keep the *F_ROH_* of this population at a low level, care should be taken to use boars and sows of different families for breeding to avoid inbreeding in the follow-up work. In addition, The genetic differences among individuals in the population should be maintained so that the combined average efficacy can be demonstrated when used as an animal model in various drug studies. Also, certain genetic exchanges with other Rongchang pig populations should be carried out to prevent the population from entering reproductive isolation without introducing other breeds and internal breeding.

## CONCLUSION

Our study on Rongchang pigs revealed that the population had a small effective population size. However, the level of heterozygosity in them was somewhat low. This suggests that we should use measures to control the level of inbreeding, such as using boars and sows from different families for breeding or introducing germplasm resources from other Rongchang pig populations appropriately, to improve N_e_ and prevent the inbreeding coefficient from continuing to increase between generations. The results of our genetic clustering analysis indicated that we should pay attention to scientific selection and optimize the population structure in the future breeding conservation process. In addition, calculating the ROH of each individual gives information on the inbreeding level of the population, so that suitable individuals can be selected for breeding. Overall, these results provide basic data to support the development of Rongchang pig breeding program, the establishment of SPF Rongchang pig closed herd and its experimental utilization.

## SUPPLEMENTARY MATERIAL

**Supplementary Table S1.** Estimation of the degree of inbreeding based on runs of homozygosity in conserved populations

## References

[1] Ma X, Yi H (2022). BMP15 regulates FSHR through TGF-β receptor II and SMAD4 signaling in prepubertal ovary of Rongchang pigs. Res Vet Sci.

[2] Chen X, Zhao C, Dou M (2020). Deciphering the miRNA transcriptome of Rongchang pig longissimus dorsi at weaning and slaughter time points. J Anim Physiol Anim Nutr (Berl).

[3] Gan L, Xie L, Zuo F, Xiang Z, He N (2015). Transcriptomic analysis of Rongchang pig brains and livers. Gene.

[4] Liu H, Hou C, Li N (2019). Microbial and metabolic alterations in gut microbiota of sows during pregnancy and lactation. FASEB J.

[5] Gerdts V, Wilson HL, Meurens F (2015). Large animal models for vaccine development and testing. ILAR J.

[6] Qi R, Sun J, Qiu X (2021). The intestinal microbiota contributes to the growth and physiological state of muscle tissue in piglets. Sci Rep.

[7] Chen L, Guo W, Ren L (2016). A de novo silencer causes elimination of MITF-M expression and profound hearing loss in pigs. BMC Biol.

[8] Gao X, Nowak-Imialek M, Chen X (2019). Establishment of porcine and human expanded potential stem cells. Nat Cell Biol.

[9] Oldenbroek JK (2021). The use of genomic information for the conservation of animal genetic diversity. Animals (Basel).

[10] Oldenbroek JK (2019). Genetic diversity in dairy cattle: Variation within and across breeds. Advances in breeding of dairy cattle.

[11] Denman A (1983). Molecular cloning: a laboratory manual. Immunology.

[12] Li H, Zhang J, Wang L A fully integrated continuous-time 50-Hz notch filter with center frequency tunability.

[13] Purcell S, Neale B, Todd-Brown K (2007). PLINK: a tool set for whole-genome association and population-based linkage analyses. Am J Hum Genet.

[14] Barbato M, OrozcoterWengel P, Tapio M, Bruford MW (2015). SNeP: a tool to estimate trends in recent effective population size trajectories using genome-wide SNP data. Front Genet.

[15] Sun H, Wang Z, Zhang Z (2018). Genomic signatures reveal selection of characteristics within and between Meishan pig populations. Anim Genet.

[16] Herrero-Medrano JM, Megens HJ, Groenen MA (2013). Conservation genomic analysis of domestic and wild pig populations from the Iberian Peninsula. BMC Genet.

[17] Sved JA (1971). Linkage disequilibrium and homozygosity of chromosome segments in finite populations. Theor Popul Biol.

[18] VanRaden PM (2008). Efficient methods to compute genomic predictions. J Dairy Sci.

[19] Gu Z (2022). Complex heatmap visualization. iMeta.

[20] Kumar S, Stecher G, Li M, Knyaz C, Tamura K (2018). MEGA X: Molecular evolutionary genetics analysis across computing platforms. Mol Biol Evol.

[21] Silio L, Rodriguez MC, Fernandez A (2013). Measuring inbreeding and inbreeding depression on pig growth from pedigree or SNP-derived metrics. J Anim Breed Genet.

[22] McQuillan R, Leutenegger AL, Abdel-Rahman R (2008). Runs of homozygosity in European populations. Am J Hum Genet.

[23] Welsh CS, Stewart TS, Schwab C, Blackburn HD (2010). Pedigree analysis of 5 swine breeds in the United States and the implications for genetic conservation. J Anim Sci.

[24] Tang GQ, Xue J, Lian MJ (2013). Inbreeding and genetic diversity in three imported Swine breeds in china using pedigree data. Asian-Australas J Anim Sci.

[25] Melka M, Schenkel F (2010). Analysis of genetic diversity in four Canadian swine breeds using pedigree data. Can J Anim Sci.

[26] Meuwissen TH, Woolliams JA (1994). Effective sizes of livestock populations to prevent a decline in fitness. Theor Appl Genet.

[27] Liu B, Tao W, Feng D (2022). Revealing genetic diversity and population structure of endangered altay white-headed cattle population using 100 k SNP markers. Animals (Basel).

[28] Liu B, Shen L, Guo Z (2021). Single nucleotide polymorphism-based analysis of the genetic structure of Liangshan pig population. Anim Biosci.

[29] Huang M, Zhang H, Wu ZP (2021). Whole-genome resequencing reveals genetic structure and introgression in Pudong White pigs. Animal.

[30] Krupa E, Moravcikova N, Krupova Z, Zakova E (2021). Assessment of the Genetic diversity of a local pig breed using pedigree and SNP data. Genes (Basel).

[31] Dobrzański J, Calik J, Krawczyk J, Szwaczkowski T (2019). Conservation of goose genetic resources in Poland - past and present status. World’s Poult Sci J.

[32] de Araujo Neto FR, Vieira DA, Santos DJA (2020). Population structure of Simmental beef cattle using pedigree analysis. Trop Anim Health Prod.

[33] Jarnecka O, Bauer EA, Jagusiak W (2021). Pedigree analysis in the Polish Red cattle population. Animal.

[34] Wang J (2016). Pedigrees or markers: Which are better in estimating relatedness and inbreeding coefficient?. Theor Popul Biol.

[35] Medugorac I, Medugorac A, Russ I (2009). Genetic diversity of European cattle breeds highlights the conservation value of traditional unselected breeds with high effective population size. Mol Ecol.

[36] Zanella R, Peixoto JO, Cardoso FF (2016). Genetic diversity analysis of two commercial breeds of pigs using genomic and pedigree data. Genet Sel Evol.

[37] Purfield DC, Berry DP, McParland S, Bradley DG (2012). Runs of homozygosity and population history in cattle. BMC Genet.

[38] Wang Y, Zhao X, Wang C (2021). High-density single nucleotide polymorphism chip-based conservation genetic analysis of indigenous pig breeds from Shandong Province, China. Anim Biosci.

[39] Peripolli E, Munari DP, Silva MVGB (2017). Runs of homozygosity: current knowledge and applications in livestock. Anim Genet.

