# Supplementary material for: Characterization analysis of Rongchang pig population based on the Zhongxin-1 Porcine Breeding Array PLUS
Source: Anim Biosci. 2023 Jun 26;36(10):1508–16. doi: 10.5713/ab.23.0049 (PMC10475371; doi:10.5713/ab.23.0049)
Supplement: Supplementary file 1 [file ab-23-0049-Supplementary-Table-1.pdf]

Supplementary Table 1. Estimation of the degree of inbreeding based on runs of homozygosity in conserved populations

Sample name	Number of runs of homozygosity	Total length of runs of	Inbreeding coefficient
611	44	187390	0.0765
18033	48	224576	0.0916
086-085	42	185132	0.0755
7628	51	393163	0.1604
42052	45	193181	0.0788
034-095	58	240588	0.0982
006-094	43	194579	0.0794
22091	49	281938	0.115
16020	48	347500	0.1418
089-091	44	225630	0.0921
057-032	47	275160	0.1123
092-060	36	191702	0.0782
7677	48	255081	0.1041
32088	39	176349	0.072
028-099	56	233055	0.0951
41076	48	240230	0.098
8075	38	148512	0.0606
49056	39	223502	0.0912
44062	53	279127	0.1139
038-055	40	223403	0.0912
25063	52	204879	0.0836
23083	49	201100	0.0821
21059	51	265426	0.1083
064-007	59	262780	0.1072
041-098	56	233388	0.0952
24049	45	186876	0.0763
43045	45	206479	0.0843
30025	46	224398	0.0916
37	51	271416	0.1107
47019	53	201887	0.0824
31065	37	147235	0.0601
10036	57	423101	0.1726
612	45	192362	0.0785
15068	46	202136	0.0825
57626	48	213879	0.0873
28099	54	237210	0.0968
10072	49	243233	0.0992
12	51	198863	0.0811
021-060	45	177805	0.0726
26067	56	344867	0.1407
071-072	47	184416	0.0752
33090	53	240967	0.0983
613	44	198773	0.0811
19024	49	210531	0.0859
051-077	47	178772	0.0729
614	43	237148	0.0968
610	42	197762	0.0807
7601-79	51	301153	0.1229
27648	45	247517	0.101

627685	43	188798	0.077
661	46	248886	0.1016
044-7607	44	192930	0.0787
084-096	47	190145	0.0776
011-004	48	231913	0.0946
